# Early discharge and international air transport of a traveler with exacerbating comorbidities and pneumomediastinum: a case report

**DOI:** 10.1186/s41182-025-00684-x

**Published:** 2025-01-31

**Authors:** Miho Akimoto, Soichiro Saeki, Yuki Kiyomoto, Hirosane Takeshima, Naofumi Higuchi, Takako Mori, Yasuyo Osanai, Chihaya Hinohara, Takeshi Inagaki

**Affiliations:** 1 Department of General Internal Medicine, Center Hospital of the National Center for Global Health and Medicine, 1-21-1 Toyama, Shinjuku, Tokyo Japan; 2 International Health Care Center, Center Hospital of the National Center for Global Health and Medicine, 1-21-1 Toyama, Shinjuku, Tokyo 162-8655 Japan; 3 Department of Emergency Medicine and Critical Care, Center Hospital of the National Center for Global Health and Medicine, 1-21-1 Toyama, Shinjuku, Tokyo Japan; 4 Department of Nursing, Center Hospital of the National Center for Global Health and Medicine, 1-21-1 Toyama, Shinjuku, Tokyo Japan

**Keywords:** International transfer, Mediastinal emphysema, Bacteremia, PICC line, Medical clearance for air travel

## Abstract

**Background:**

Air transport for medically complex patients presents unique challenges, particularly without specific guidelines for conditions such as pneumomediastinum or medical devices like peripherally inserted central catheters (PICC lines). Although organizations such as the Aerospace Medical Association (AsMA) and the International Air Transport Association (IATA) provide general recommendations for medical air travel, these guidelines often lack the precision required to address such complex clinical scenarios. Consequently, healthcare teams frequently face difficult decisions under constraints of time and resources, navigating the interplay of patient safety, autonomy, and logistical considerations.

**Case presentation:**

This case involves a 30-year-old American woman with type 2 diabetes, obesity, and a recent history of pancreaticoduodenectomy, who was hospitalized in Japan with cellulitis and incidentally diagnosed with pneumomediastinum. She was treated with intravenous antibiotics and central venous nutrition administered via a PICC line. However, she requested an early discharge to return to the United States for family and financial reasons. Her travel insurer declined coverage, citing potential risks associated with pneumomediastinum. Ultimately, the patient discharged herself against medical advice; the PICC line was removed, and she transitioned to oral antibiotics for her journey home.

**Conclusion:**

This case highlights the complexities of patient preferences, medical risks, and insurance limitations when evaluating air travel safety. The absence of specific guidelines for conditions such as pneumomediastinum and the use of medical devices highlights the need for condition-specific protocols. Effective communication and customized documentation, including modifications to the "Against Medical Advice" form proved essential in addressing both patient autonomy and the responsibilities of healthcare providers.

## Background

Air transport of medically complex patients presents unique challenges, particularly without definitive guidelines. While the Aerospace Medical Association (AsMA) and the International Air Transport Association (IATA) have established general guidelines [1, 2], they frequently lack specific criteria for individual diseases or medical devices. This ambiguity becomes particularly problematic when addressing conditions such as pneumomediastinum or the presence of peripherally inserted central catheters (PICC lines), where the risks associated with fluctuations in cabin pressure and potential complications are insufficiently delineated.

We present a case involving a traveler who required medical evacuation after experiencing pneumomediastinum and necessitated a PICC line for treatment.

## Case report

An American woman in her 30s, with a history of type 2 diabetes and obesity, presented with severe bilateral leg pain accompanied by exudate and impaired mobility in Japan. She initially sought care at a local clinic, where the attending physician, following an initial assessment, deemed her condition to require urgent intervention. Consequently, an emergency transfer was arranged, and the patient was transported by ambulance to our hospital (day X). She had previously traveled to Japan with her friend and 6-year-old son on a 13-h flight (day X-3). She developed erythema, vesicles, and erosions on both lower extremities and decubitus ulceration on the buttocks (day X-2). She underwent biliopancreatic diversion surgery in the United States and developed chronic edema and dermatitis attributed to trace element deficiency post-operatively.

**Fig. 1:**
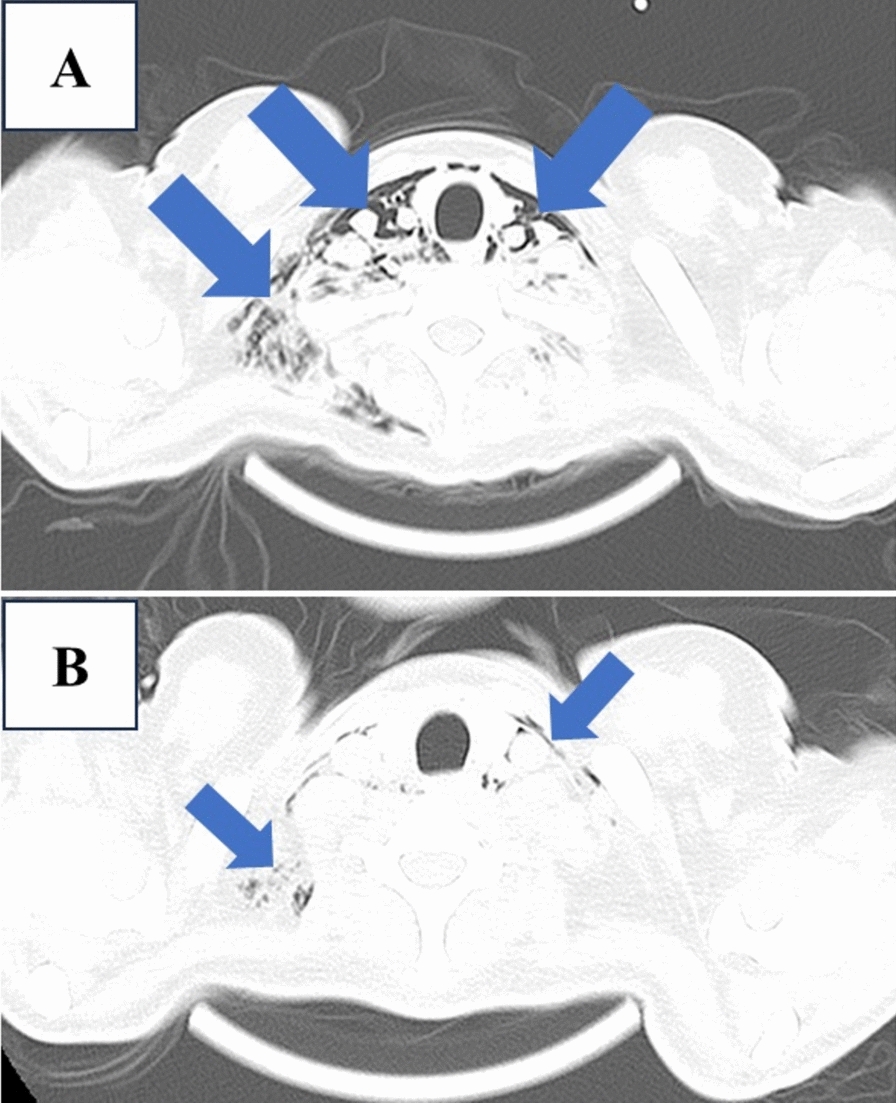
**CT of the pneumomediastinum**. The CT graphics of the patient’s mediastinum (indicated by the arrows) on day X (date of admission, **A**) and on day X + 5 (**B**). Pneumomediastinum improved with conservative treatment. *CT* computed tomography

Admission computed tomography (CT) incidentally revealed mild pneumomediastinum of unknown etiology (Fig. 1a). Upon admission, the patient reported no posterior sternal pain, dyspnea, or voice changes. Physical examination was notable only for subcutaneous crepitation in the neck. While no typical risk factors for spontaneous pneumomediastinum, such as coughing, sneezing, vomiting, or recent strenuous activity, were identified, it was noted that the patient had visited a theme park the previous day and may have screamed loudly while on a ride. The patient’s medical history was negative for asthma or cystic lung disease. Given the patient’s stable vital signs, a conservative management approach was adopted.

Blood cultures upon admission identified multiple pathogens, including methicillin-susceptible *Staphylococcus aureus* and Gram-negative rods, requiring intravenous antibiotics and daily wound care. Due to significant edema, a PICC line was placed on day X + 3 for central venous nutrition and trace element supplementation. We recommended a 14-day course of intravenous antimicrobial therapy for bacteremia. However, the patient, feeling mentally unstable and eager to see her child in the United States, requested an early discharge. Although the patient had procured travel insurance covering pre-existing conditions, upon discussion with the medical team, the insurance company denied coverage for premature discharge, citing the risk of exacerbation of her pneumomediastinum from aircraft transport. Consequently, she decided against an early return home (day X + 6).

A subsequent meeting with the insurance company (day X + 9) was held to convey that early discharge was medically feasible; however, they recommended completing treatment in Japan. Financial constraints ultimately prevented her from affording a medical team for the flight. On day X + 10, facing the prospect of extended hospitalization, she ultimately opted to discharge herself against the hospital’s medical advice. The PICC was removed before her flight on the discharge day, and she commenced oral antibiotics simultaneously. Following discharge from our facility, the patient successfully completed her flight to the United States without any reported complications. Correspondence with her primary care physician confirmed that she was hospitalized and received appropriate medical care upon arrival. Subsequent imaging conducted in the United States demonstrated no progression of the pneumomediastinum.

## Conclusions and discussion

This case highlights the complexity of determining when to discharge and permit air travel following unexpected hospitalization due to an exacerbation of a pre-existing condition.

The patient presented multiple medical issues; however, the cellulitis and decubitus ulceration improved with inpatient care, leaving pneumomediastinum as the primary concern. The insurance company contended that patients with pneumomediastinum should adhere to pneumothorax management guidelines and fly after full resolution. While pneumomediastinum typically does not worsen during air travel [3, 4], there have been fatalities associated with underlying lung disease [5, 6]. Nonetheless, as the patient had no underlying pulmonary conditions contributing to her pneumomediastinum and exhibited no exacerbations following conservative management (Fig. 1B), she was ultimately assessed as fit to fly.

The second issue involved PICC. Safety considerations included alterations in cabin pressure, embolization, and line disconnection risk. Given the likelihood of no medical escort, safety could not be entirely guaranteed. However, with negative blood cultures and partial oral intake capability, the PICC was removed, and the patient was cleared for flight with an oral antimicrobial switch. Neither the AsMA nor the IATA guidelines explicitly address flight suitability for patients with these conditions and devices, necessitating a case-by-case assessment based on individual circumstances.

It was concluded that the patient would require hospitalization after return, and air transport was feasible. Physicians must complete a clearance document [7] certifying that the patient is medically “fit to fly.” However, this does not confirm the patient’s readiness for discharge. Although we recommended a 14-day course of intravenous antimicrobial treatment, the patient desired earlier discharge. She was asked to sign an “Against Medical Advice (AMA)” form to acknowledge the risks of early discharge, noting that the discharge decision contradicted the treatment plan. The patient insisted that the issue was not the discharge itself but the flight without a medical escort, worrying that the documentation might affect insurance coverage. Typically, patients sign AMA forms for discharge, so we modified the wording to clarify it as a risk explanation regarding the flight without a medical escort. The AMA form does not shield against litigation but aims to ensure adequate patient understanding and documentation [8] (Table 1). Effective communication facilitated a mutually acceptable documentation process, respecting the patient's wishes while fulfilling the medical team's obligations.

**Table 1:** Comparison of fit to fly and against medical advice forms

Documents	Purpose	Requirement	Flight	Discharge	Insurance coverage
Fit to fly	Advising whether a flight is possible	The airline	○	× 〜○	No relation
AMA	Confirming the patient is discharged on the patient’s responsibility	The hospital	No relation	○	No relation

Whether the flight/discharge/Insurance coverage will be allowed based on these documents

*AMA* against medical advice; ○ Possible; × Not possible

The patient negotiated with the insurance company to cover her medical expenses in Japan, but ultimately, none of the costs were covered. Insurance coverage for pre-existing conditions does not guarantee coverage for all associated medical costs. The rationale behind the coverage denial was not disclosed. Until 2010, no reports of AMA documents led directly to insurance coverage denials based on surveys confined to the United States [9]. Consequently, attributing the denial exclusively to the patient's early flight was unsubstantiated. In severe cases requiring international transport, securing insurance coverage remains a significant concern due to high medical expenses [10].

In conclusion, patients seeking early discharge by air transport should first undergo a medical feasibility assessment. Generally, flight clearance decisions are based on a patient’s discharge readiness; however, flights may be permitted for international transport even if the discharge is not advised, considering hospitalization requirements upon return. These cases reveal the high costs of medical care and documentation challenges that can arise when there is a disconnect between medical providers and patients. Medical personnel must navigate these unique challenges of early international transport and maintain open communication with patients.

## Data Availability

No datasets were generated or analysed during the current study.

## Abbreviations

AsMA: Aerospace Medical Association
IATA: International Air Transport Association
IV: Intravenous
PO: Per os
AMA: Against medical advice
PICC: peripherally inserted central catheters
